# Protection against H9N2 challenge in chickens via *Salmonella* with a *focA* mutation delivered by a dendritic cell-targeted avian influenza virus NA protein tetramer

**DOI:** 10.1128/spectrum.01421-25

**Published:** 2025-10-27

**Authors:** Chongbo Ge, Yan Sun, Qiyu Guo, Gerui Zhang, Futing Jia, Tongyu Zhang, Yawen Tian, Wenfeng Wang, Yingkai He, Tianrui Yang, Yupeng Gao, Yuxi Zhang, Yuhang Zhang, Mingyue Wang, Jingshuo Gong, Haibin Huang, Zhannan Wang, Chunfeng Wang, Yanlong Jiang

**Affiliations:** 1 College of Animal Medicine, Jilin Provincial Engineering Research Center of Animal Probiotics, Jilin Provincial Key Laboratory of Animal Microecology and Healthy Breeding, Engineering Research Center of Microecological Vaccines (Drugs) for Major Animal Diseases, Ministry of Education, Jilin Agricultural University 85112, Changchun, China; 2 College of Modern Agriculture, Changchun Polytechnic University, Changchun, China; Institute of Microbiology Chinese Academy of Sciences, Beijing, China

**Keywords:** chicken DC targeting, *focA*-deleted *Salmonella*, tetramer-NA, H9N2 vaccine

## Abstract

Vaccination is considered one of the most effective methods for preventing avian influenza. The influenza neuraminidase (NA) enzyme is a promising candidate for a conserved influenza vaccine, offering long-term and effective cross-protection against the disease. The aim of this study is to develop a *Salmonella*-vectored vaccine with strong protective efficacy against H9N2 avian influenza virus. We utilized the antibiotic-free balanced-lethal system of regulated delayed-lysis *Salmonella* (χ11218) as the starting strain to construct a *focA* gene-deleted *Salmonella* (χYL57) strain. To optimize the vaccine design, we fused a tetravalent NA protein (tetramer-NA) with a chicken dendritic cell-targeting peptide (chDCpep) and delivered it via χ11218 or χYL57, yielding the candidate vaccine strains S323 or ΔS323, respectively. The results revealed that the successful synthesis of tetramer-NA proteins could be detected using nonreducing western blotting and that the presence of chDCpep could dramatically increase the maturation of chicken bone marrow-derived dendritic cells. The *focA* deletion enhanced bacterial escape from macrophages *in vitro* and bacterial colonization *in vivo*. After oral immunization, the combination of chDCpep and *focA* gene deletion efficiently improved the humoral and mucosal immune responses. Additionally, CD4 and CD8 T cell proliferation in the chicken spleen significantly increased, with elevated intracellular interleukin-4 and interferon-γ mRNA levels. After the virus challenge, the virus titers in the lungs and trachea of the chickens appeared to significantly decrease, as did the production of the inflammatory cytokines IL-6 and IL-1β. Moreover, improved body weight gain and histological lesions were observed. In summary, the combination of chDCpep and *focA* deletion provides a novel option for the design of a *Salmonella*-based oral vaccine against avian influenza virus infection.

**IMPORTANCE:**

A *focA* gene deletion was introduced into *Salmonella* with delayed lysis, resulting in increased cellular escape and colonization. Afterward, a chicken DC-targeting peptide was ligated to the tetramer neuraminidase protein of the H9N2 influenza virus. The combination of the *focA* gene mutation and chicken DC targeting effectively enhanced the humoral, cellular, and mucosal immune responses, yielding improved protection against H9N2 challenge in chickens.

## INTRODUCTION

Avian influenza (AI) is a severe respiratory disease in poultry caused by the avian influenza virus (AIV). It includes highly pathogenic avian influenza (H5N1), low-pathogenicity avian influenza (H9N2), and nonpathogenic avian pneumonia. This disease is widely prevalent globally, leading to a range of symptoms in birds and poultry and even posing a threat to human life (1). AIV belongs to the family Orthomyxoviridae and the genus Influenza A virus and is a single-stranded negative-sense RNA virus (2). AIV can be classified into different subtypes on the basis of surface glycoproteins, such as hemagglutinin (HA) and neuraminidase (NA). In recent decades, several subtypes of AIV, including H9N2, H5N1, and H7N9, have become prevalent (3). Among them, the H9N2 subtype avian influenza virus was first discovered in turkeys in Wisconsin in 1966 and emerged in Guangdong Province, China, in 1992. It is a low pathogenic avian influenza virus that primarily infects and spreads through the respiratory mucosa, leading to seasonal respiratory symptoms (4). Initially, H9N2 infected only turkeys, but owing to its segmented genome and error-prone RNA polymerase, H9N2 is prone to gene mutation and rearrangement, resulting in the expansion of its host range of H9N2, including chickens, ducks, pigs, and even humans (5). Furthermore, H9N2 is also a potential gene donor for other influenza viruses, such as H7N9, which is highly pathogenic to humans, increasing the likelihood of viral mutation and reassortment (6). Currently, vaccination is the most effective method for preventing influenza spread, but owing to the high mutation rate of avian influenza viruses and the diversity of circulating subtypes, existing influenza vaccines are not fully effective at preventing influenza outbreaks (7).

In the development of avian influenza vaccines, viral HA protein has been used as the primary target because anti-HA antibodies usually neutralize and protect host cells from viral attachment and entry (8). NA, the second most abundant glycoprotein on the virus surface, is a tetramer with enzymatic activity that plays a crucial role in the viral budding process (9). Notably, a total of 11 different subtypes of NA proteins have been identified, of which N10 and N11 NA are unique to bats (10). NA-induced-specific antibodies promote immune efficacy by limiting viral replication and disease severity (11) and function as receptor-destroying enzymes, cleaving sialic acid receptors, which are essential for viral release and dissemination (12). Furthermore, the mutation rate of the NA protein is low, and the NA protein can induce cross-protective immune responses between species, making it an ideal target for conserved influenza vaccines (13). Currently, existing vaccine-induced HA antibodies have been extensively studied, generally providing protection by preventing homologous viruses from attaching to and fusing with host cells. Research has indicated that NA antibodies can provide varying degrees of disease protection in the absence of well-matched HA antibodies (14). Therefore, there is an urgent need for AIV-H9N2 vaccines, and NA from H9N2 presents significant potential as an antigenic target for vaccine development. Currently, challenges in designing vaccines targeting the NA protein still exist. The anti-NA immunity induced by existing influenza vaccines is relatively weak, primarily because of the low abundance and poor stability of the NA antigen (15). Notably, compared with monomeric NA proteins, tetrameric NA with a natural conformation was reported to be required to induce protective anti-NA immunity in BALB/c mouse models (16). Some well-known tetramerization domains, such as the 45-amino acid human vasodilator-stimulated phosphoprotein (hVASP) (17), have shown great potential in stabilizing the NA head region for vaccine purposes (18).

Dendritic cells (DCs) are found primarily in mucosal and lymphoid tissues and are important members of the innate and adaptive immune system. DCs play a crucial role in initiating adaptive immunity and effectively stimulate adaptive immune responses to combat pathogens (19). DCs are also the primary antigen-presenting cells and play a significant role in antigen capture, processing, and presentation (20). In terms of vaccine design, DC-targeting strategies have shown great promise for enhancing vaccine efficacy through the use of monoclonal antibodies (21), nanobodies (22), or DC-targeting peptides (DCpep) (23). DCpep is a short DC-targeting peptide that specifically binds to receptors on dendritic cells, facilitating the rapid and effective release of antigens recognizable by dendritic cells without affecting cell phenotype or function, thereby more effectively activating specific T cells and yielding enhanced humoral and cellular immune responses in both mammalian (24) and chicken studies (23). For example, Xiaohong Xu et al. reported the modification of Newcastle disease virus hemagglutinin-neuraminidase and H9N2 HA virus-like particles with DCpep, which led to enhanced activation of chicken DCs *in vivo* and promoted sIgA secretion and splenic T cell differentiation, efficiently inhibiting H9N2 virus shedding when administered intranasally (25).

*Salmonella* has been widely employed in the field of poultry vaccine studies because of its convenient application by oral immunization and ease of manipulation using genetic engineering. In particular, the regulated delayed-lysis/attenuation/expression *Salmonella* vectors designed by Dr. Roy Curtiss III represent some of the most advanced bacterial vectors for vaccine design because they can appear similar to wild-type *Salmonella,* which guarantees efficient colonization *in vivo*, whereas the bacteria could gradually attenuate, synthesize foreign antigens, and lyse after colonization (26). Notably, only a small portion of *Salmonella* escape and are transferred to host intestinal epithelial cells for colonization (27). On the other hand, Kammel et al. reported that after the *Salmonella focA* gene was deleted, macrophage phagocytosis disrupted the homeostasis of macrophages, and immune cells accelerated the frequency of “foreign” metabolism, allowing more *Salmonella* to escape macrophages and spread to other organs, such as the liver and spleen (28, 29). This finding led us to determine whether the deletion of the *focA* gene in our *Salmonella* vector could benefit the immune response in chickens.

In this study, the extracellular region of the NA antigen from the H9N2 subtype AIV was ligated to the tetramer domain of hVASP and conjugated with chicken DCpep. In addition, a *focA* gene-deleted *Salmonella* strain was generated as a vector to determine whether it could enhance the humoral immune response in a chicken study.

## MATERIALS AND METHODS

### Bacterial strains and virus used

The bacterial strains, plasmids, and cell lines used in this study are listed in Table 1. The bacterial strains were cultured using Luria-Bertani (LB) medium at a constant temperature of 37°C, and 50 µg/mL 2,6-diaminopimelic acid (DAP) (Sigma) and 0.1% arabinose (Sigma) were provided when necessary. For the construction of the suicide plasmid, 25 µg/mL chloramphenicol was used. The H9N2 subtype AIV isolate G57 (A/Chicken/Jilin/DH109/2012) was generously provided by Yanlong Cong from Jilin University and preserved in our laboratory. The virus was cultured in the allantoic cavity of 9- to 11-day-old specific pathogen free eggs provided by the Harbin Veterinary Research Institute (Harbin, China). The virus was determined to have an egg infective dose 50% (EID_50_) of 10^7^ and was used in the challenge experiment of chicks.

**TABLE 1:** Bacterial strains, plasmids, and cell lines

Materials	Description	Source
Bacteria strains		
χ6212	*Escherichia coli* host strain with *asd* mutation	Roy Curtiss III University of Florida
χ11218	Regulated delayed-lysis *Salmonella*	Roy Curtiss III University of Florida
χYL57	Regulating delayed lysis of *focA* gene-deleted *Salmonella*	This study
Top10	*E. coli* host strain for regular cloning	Kangti, China
χ7232	*E. coli* host strain for suicide plasmid construction	Roy Curtiss III University of Florida
χ7213	*E. coli* host strain for bacterial conjugation	Roy Curtiss III University of Florida
Plasmids		
pRE112	Suicide plasmid for *focA* gene deletion	Addgene
pYL305	pRE112-based suicide plasmid for *Salmonella focA* gene	This study
pYL46	Eukaryotic expression plasmid with a minicircle DNA design, CMV promoter	Lab stock
pYL187	Synthesized complete NA gene of G57 genotype H9N2 influenza virus, codon optimized for expression in chicken	Genewiz, Suzhou, China
pYL310	Synthesized hVASP tetramer region, codon optimized for expression in chicken	Genewiz, Suzhou, China
pYL313	hVASP tetramer region fused with extracellular region of NA (32–466 aa)	This study
pYL314	CMV promoter, hVASP tetramer region fused with extracellular region of NA (32–466 aa)	This study
pYL323	CMV promoter, hVASP tetramer region fused with extracellular region of NA (32–466 aa) and chicken DCs targeting peptide	This study
Cell lines		
HEK 293T	Human embryonic kidney cell	Meilun, China
RAW264.7	Mouse mononuclear macrophage leukemia cells	Lab stock
chBM-DC	Chicken bone marrow-derived dendritic cells	This study

### Construction of plasmids

**Fig 1:**
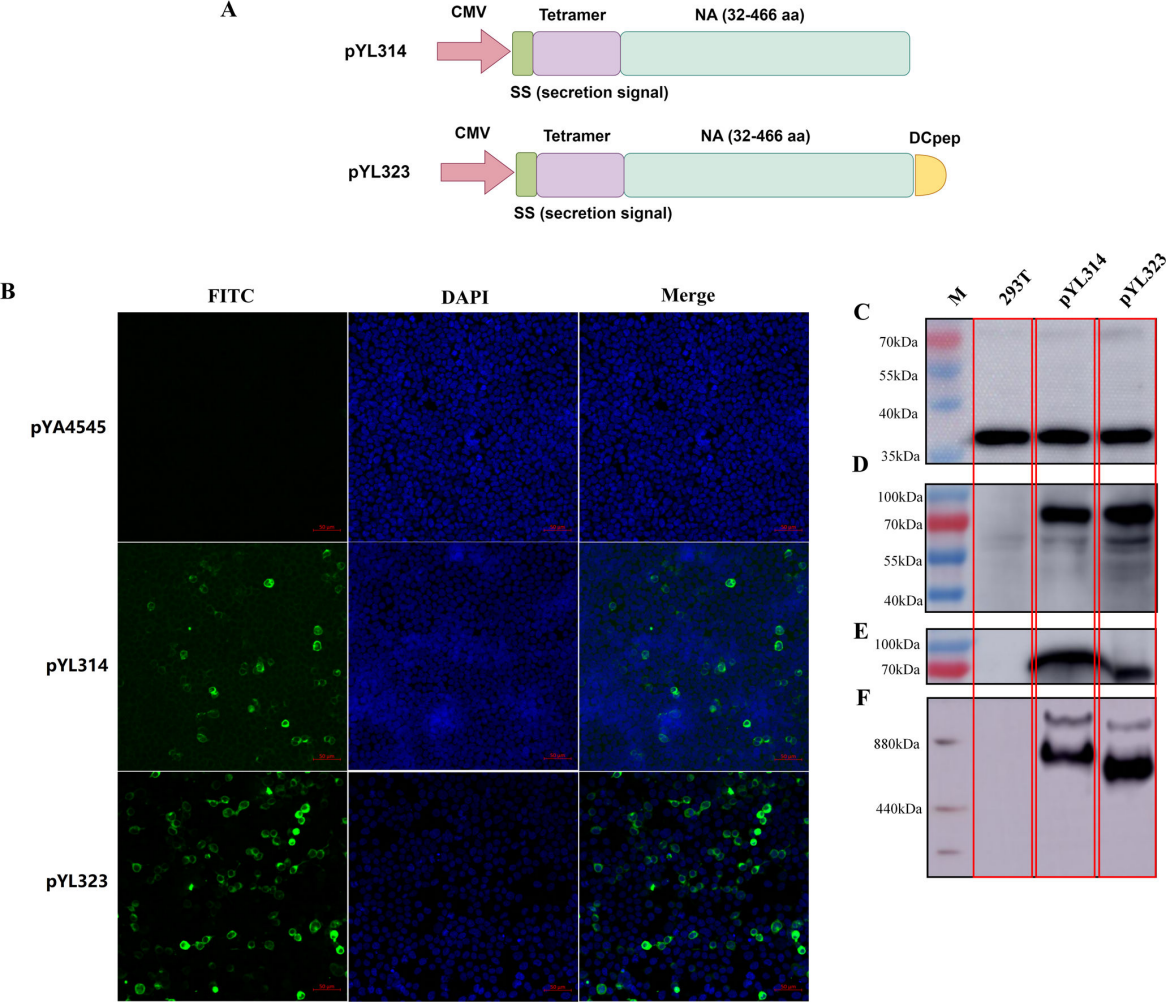
Plasmid construction and protein synthesis. The extracellular region of H9N2 NA was ligated with the tetramer sequence and inserted after the CMV promoter, with or without a chicken DCpep sequence, yielding pYL323 and pYL314, respectively (**A**). The two plasmids and the parental plasmid pYA4545 (as a negative control) were transfected into 293T cells, and NA protein synthesis was confirmed with immunofluorescence analysis using an NA-specific antibody and an fluorescein isothiocyanate (FITC)-labeled secondary antibody (**B**). Proteins extracted from transfected cells were also subjected to western blotting using antibodies targeting GAPDH (**C**) and NA (**D**). In addition, the supernatants of transfected 293T cells were collected and analyzed with reducing (**E**) and nonreducing (**F**) western blotting using an NA-specific antibody as the primary antibody.

The primers used in this study are shown in Table 2. The plasmid encoding the hVASP tetramer region was fused with a secretion signal sequence (SS), codon-optimized for chicken preference, and synthesized by Genewiz (Suzhou, China); this plasmid is named pYL310. Using the plasmid pYL187 (chicken NA) as a template, the extracellular domain of the NA protein (32–466 aa) was amplified by 187NA-F-SpeI/187NA-R-BamHI, digested with BamHI/SpeI, and then ligated with the plasmid pYL310 digested with the same enzymes. The digested products were then ligated using T4 DNA ligase and transfected into Top10 competent cells to obtain pYL313 (Fig. S1). The pYL313 and eukaryotic expression vector pYL46 were then digested using KpnI/NotI, ligated together using T4 DNA ligase, and electroporated into *Escherichia coli* χ6212 competent cells to obtain pYL314 (Fig. 1A). The primers 313DC-NA-F-KpnI/313DC-NA-R-NotI were subsequently used to amplify the NA-DCpep fragment from pYL314, including the extracellular domain of the NA protein and an additional chicken DCpep sequence (SPHLHTSSPWER)(23). The fragment was then digested with KpnI/NotI and ligated with pYL46 as described above, yielding pYL323 (Fig. 1A).

**TABLE 2:** Primers for constructing plasmids

Primers	Sequences
187NA-F-SpeI	GGACTAGTACCATGACCCTGCACTTC
187NA-R-BamHI	CGCGGATCCTCACTTGTCGTCGTCGTC
313DC-NA-F-Kpnl	GGTACCAGGAGCCGCCACCATGGA
313DC-NA-R-NotI	CCGCGGCCGCTCATCTCTCCCATGGAGA
focA-up-F2-SmaI	TATCCCGGGTAGTCACCGATGATACGGC
focA-up-R2	GCATGCCCTAAGCTGGTACCTCTGTTGATGCCCATTACAC
focA-down-F2	GGTACCAGCTTAGGGCATGCGGGTTGTCAGCTTTCACAC
focA-down-R2-XbaI	CGCTCTAGATGGACTACCGGCTGTCGAC

### Determination of protein synthesis

Briefly, 293T cells were cultured in Dulbecco's Modified Eagle Medium (DMEM) supplemented with 10% fetal bovine serum, 50 µg/mL streptomycin, and 50 U/mL penicillin in a cell culture plate with a climbing sheet for 24 h, after which the purified plasmids (pYL314 and pYL323) and pYA4545 were transformed into 293T cells using PolyJet transfection reagent (SignaGen) for 48 h to evaluate their expression.

To evaluate the synthesis of recombinant protein, the cells were fixed with 4% paraformaldehyde for 20 min, after which the cell membrane was perforated with 0.5% Triton X-100 solution. The cells were then incubated in a 2% bovine serum albumin (BSA) solution for blocking at 4°C overnight. Then, the cells were incubated with the primary antibody against the NA-specific antibody (Sino Biological, China), followed by incubation with the corresponding FITC-conjugated secondary antibody (TransGen Biotech, China). After being fully washed, the nuclei were stained with 4',6-diamidino-2-phenylindole (DAPI) (Beyotime Biotechnology, China) and observed under a fluorescence microscope.

Further evaluation of recombinant protein expression using western blotting was also conducted. In detail, the culture supernatants were collected and concentrated 10 times using an ultrafiltration concentration tube (Millipore), whereas the remaining cells were subjected to radioimmunoprecipitation assay (RIPA) lysis (Beyotime Biotechnology, China) to collect whole-cell proteins (proteins from plasmids pYL314 and pYL323, subsequently referred to as P314 and P323, respectively). The levels of P314 and P323 in the supernatants and cell samples were then analyzed using western blotting. After separation via SDS-PAGE, the proteins were transferred onto a polyvinylidene fluoride membrane. After the membranes were blocked with tris-buffered saline with tween (TBST) solution containing 5% skim milk, rabbit polyclonal anti-NA antibody (Sino Biological, China) and horseradish peroxidase (HRP)-conjugated goat anti-rabbit IgG antibody (TransGen Biotech, China) were used as primary and secondary antibodies, respectively, and an enhanced chemiluminescence reaction (Thermo Fisher, USA) was performed.

### Isolation of chicken bone marrow-derived dendritic cells (chBMDCs) and evaluation of the effects of purified protein stimulation on maturation

chBMDCs were isolated according to a previously described method (30) with slight modifications. In detail, 3-day-old chicks were sacrificed, and bone marrow-derived monocytes were collected from the femur. Then, the red blood cells in the cell suspension were removed using a lymphocyte separation kit (P9120; Solaibao Co., Ltd., China). Roswell Park Memorial Institute (RPMI) 1640 complete medium supplemented with recombinant chicken granulocyte-macrophage colony stimulating factor (GM-CSF) (30 ng/mL) and IL-4 (20 ng/mL) (IBI Scientific, USA) was used. The cell concentration was adjusted to 3 × 10^6^ cells/well in a six-well cell culture plate. At the same time, 2 mL of culture medium containing stimulators was added to each well. Half of the medium containing stimulators was replaced on the 1st, 3rd, and 5th days of culture, and the cell status was recorded by optical microscopy daily.

On the 7th day, all the cells were collected and subcultured in a six-well plate, with three replicates in each group. Four groups were designed, namely the negative group, positive group (lipopolysaccharide, LPS, 1 mg/mL), tetramer-NA group (protein from plasmids pYL314 and P314, 20 µg/mL), and tetramer-NA-DCpep group (protein from plasmids pYL323 and P323, 20 µg/mL). The proteins and LPS were incubated with chBMDCs for 24 h, after which total RNA was extracted and subjected to a quantitative reverse transcription polymerase chain reaction (qRT-PCR) assay targeting the maturation marker molecules CCL5, CCR7, CD83, and CD86 using the primers listed in Table 3.

**TABLE 3:** Primers for real-time PCR used in this study

Primers	Sequences
chCCL5-F	F:CCGTGTGCTGCTTCAACTAT R:CTTCCTGGTGATGAACACAACT
chCCR7	F:GCTTTCAGCCTTCCTCTCAT R:GACCTCCTTCTTCTCACACATC
chCD83	F:CTGGGAGTTGGCGACAGAATAG R:TTGAAAGGTGGTGTTGGAGAG
chCD86	F:TACCTTGGCCAGGAAAAACA R:ATACTGCCCCTCATCCACAA
chβ-actin	F:CTATCITTCACCACTTCTC R:CTCAGAGAAAGCCAGCTTAGAG
chIFN-γ	F:GTGAAGAAGGTGAAAGATATCATGGA R:GCTTTGCGCTGGATTCTCA
chIL-4	F:AACATGCGTCAGCTCCTGAAT R:TCTGCTAGGAACTTCTCCATTGAA
chH9N2-M1	F:GCAAGCGCACCAGTTGAGTAA R:GGGAATGGAGACCCAAACAA
chGAPDH	F:TGCTGCCCAGAACATCATCC R:ACGGCAGGTCAGGTCAACAA
chIL-6	F:CAAGGTGACGGAGGAGGAC R:TGGGCATCAAGGGATTTCT
chIL-1β	F:TCGGGTTGGTTGGTGATG R:TGGGCATCAAGGGCTACA

### Construction of the *focA* gene-deleted *Salmonella* strain and evaluation of its characteristics

The primers used in this study are shown in Table 2. The χ11218 genome was extracted and used as a template to amplify the target fragments focAUP and focADOWN using the primers focA-UP-F2-SmaI/focA-UP-R2 and focA-DOWN-F2/focA-DOWN-R2-XbaI, respectively. The two fragments were then fused together using the primers focA-UP-F2-SmaI/focA-DOWN-R2-XbaI and ligated with pRE112 digested with SmaI/XbaI to yield the suicide plasmid pYL305. After transformation into *E. coli* χ7213 competent cells, the strains were mixed with χ11218 and screened by two rounds of selection for *focA* gene deletion, yielding the mutant *Salmonella* strain χYL57. The pYL314 and pYL323 plasmids were transfected into the competent cells of χ11218 and χYL57 using electroporation, and the products were inoculated onto LB plates containing arabinose for cultivation. Individual colonies were selected, and the plasmids were extracted and sent for sequencing to confirm successful transfer. To determine whether the presence of the *focA* gene deletion affected the growth of the *Salmonella* strain, both strains were inoculated into fresh LB medium supplemented with 0.2% arabinose and DAP and incubated at 37°C with shaking. The optical density at 600nm‌ (OD_600_) values of both cultures were recorded every 2 h until 12 h.

Both *in vitro* and *in vivo* studies were subsequently performed to evaluate the effect of the deletion of the *focA* gene. For the *in vitro* study, macrophage RAW264.7 cells were used to determine the adhesion, invasion, intracellular proliferation, and extracellular escape ability of the Δ*focA*-attenuated *Salmonella* strain. In detail, RAW264.7 cells were plated into 24-well cell culture plates at 5 × 10^5^ cells per well for 24 h. Then, the bacterial solution was added at an multiplicity of infection (MOI) of 100:1, and three replicates were established for each group. Immediately after the bacterial solution was added, the cell culture plates were centrifuged at 1,000 rpm for 10 min at room temperature. To detect adhesion ability, the supernatants were discarded after 1 h of incubation, and the cells were lysed with 200 µL of 0.2% Triton X-100 at room temperature for 2 min, after which the surviving bacteria were counted by plating on LB agar supplemented with 0.2% arabinose and DAP. To evaluate the invasion ability of the *Salmonella* strains, the supernatants were discarded after 1 h of incubation. Then, 500 µL of complete DMEM supplemented with 100 µg/mL gentamicin was added and incubated for another hour. Then, the intracellular bacteria were counted as described above.

The proliferation and escape ability of cells with a *focA* gene deletion mutation were also determined. After 1 h of incubation with either χYL57 or χ11218, the supernatants were discarded, and complete DMEM supplemented with 0.2% Ara and DAP but without antibiotics was added. Then, the samples were incubated for another 10 h. The cells and supernatants were then collected at 1, 5, and 10 h, and the surviving bacteria were counted on LB plates supplemented with 0.2% arabinose and DAP.

In addition, to determine the *in vivo* distribution of the mutant *Salmonella* strain, 3-day-old chickens were orally immunized with the χYL57 and χ11218 strains at a dose of 1.0 × 10^9^ colony-forming unit (CFU)/0.1 mL (*n* = 3). Afterward, at days 3 and 7 post-immunization, liver and spleen samples were collected, and the colonization of *Salmonella* was evaluated by plating on SS medium plates harboring arabinose and DAP. The liver was also collected for pathological tissue analysis.

### Immunization and sample collection

A total of 105 1-day-old Hy-Line Brown hens (Changchun Academy of Agricultural Sciences, Changchun, China) were randomly divided into seven groups with 15 in each group: the control group (buffer saline with gelatin, BSG), the χ11218 with empty vector group (S4545), the vaccine group, the *Salmonella* χ11218 with tetramer-NA group (S314), the χ11218 with chicken dendritic cell-targeting peptide (chDCpep)-tetramer-NA group (S323), the *focA* gene mutant *Salmonella* χYL57 strain with chDCpep-tetramer-NA group (ΔS323), and the normal group (without immunization and challenge). The chickens were orally immunized on day 21 at a dose of 1 × 10^9^ CFU/100 µL BSG buffer when the parental H9N2 antibody titers decreased to less than 24, which was recorded as day 0. A second boost immunization was then performed 14 days later at the same dose. Notably, the inactivated vaccine (recombinant triple inactivated vaccines against Newcastle disease virus, infectious bronchitis virus, and H9 subtype avian influenza virus; Harbin Pharmaceutical Group Biological Vaccine Co., Ltd.) was injected once on day 0.

On day 24 (10 days post-second immunization, 10 dp2i), four chickens from each group were sacrificed, and samples, including serum, tracheal lavage fluid, alveolar lavage fluid, and intestinal lavage fluid, were collected to analyze humoral and mucosal immunity using enzyme-linked immunosorbent assay (ELISA). To prepare the alveolar lavage fluid, a 1 mL syringe was used to aspirate the prechilled phosphate buffer saline (PBS) and recover it after repeated aspiration within the alveoli. To prepare the trachea and intestinal lavage fluid, the trachea and intestinal samples were cut, placed in precooled PBS solution, allowed to stand at low temperature for 30 min, and then allowed to recover after shaking with a vortex oscillator for 5 min. In addition, spleen cells were isolated, and cell proliferation was determined using flow cytometry and the Cell Counting Kit-8 (CCK-8) method. The intracellular production of the cytokines IL-4 and IFN-γ in the spleen cells was also determined by qRT-PCR, and the production of cytokines in the supernatant of the cultured cells was determined using a commercial ELISA kit (Kete, Jiangsu, China).

Chickens were challenged with the H9N2 G57 serotype strain A/Chicken/Jilin/DH109/2012 (kindly provided by Professor Yanlong Cong from Jilin University) via nasal inhalation at a dose of 10^7^ EID_50_ on day 28 (14 days after the second immunization, 14 dp2i). The chickens were monitored daily for clinical signs, and body weight gains were recorded daily for 14 days. On the 5th day post-challenge (5 dpc), three chickens were randomly selected from each group, and lung and organ tissues were collected to determine the viral titer and inflammatory factor content. At the same time, pathological sections were observed to analyze the lesions. Oropharyngeal and cloacal swabs were then collected from the birds, and these samples were inoculated into 10-day-old chicken embryos to detect virus shedding on days 3, 5, 7, and 9 post-viral challenge (dpc). The samples were repeatedly freeze-thawed three times before being inoculated into 10-day-old chicken embryos, which were incubated at 37°C for 3 days, after which the allantoic fluid of the embryos was collected. The obtained allantoic fluid was then tested for viral positivity using a hemagglutination assay.

### ELISA

A 96-well ELISA plate was coated with recombinant NA protein at a concentration of 1 µg/mL at 4°C overnight. After being washed three times with phosphate buffered saline with tween (PBST), a blocking solution comprising 5% BSA was used to prevent nonspecific binding. The serum and mucosal samples were then diluted twofold at initial dilutions of 1:100 and 1:10, respectively. After incubation for 1 h at 37°C, the plates were washed three times with PBST and then incubated with biotin-labeled goat anti-chicken IgG or IgA antibody (Southern Biotechnology, Birmingham, AL) at a dilution of 1:10,000, followed by streptavidin-labeled HRP (Southern Biotechnology, Birmingham, AL) diluted to 1:3,000. Afterward, the 2,2'-azino-bis(3-ethylbenzothiazoline-6-sulfonic acid) (ABTS) substrate was added, and the results were recorded at 405 nm.

### Lymphocyte proliferation assay

The spleen lymphocytes were isolated using a chicken spleen lymphocyte isolation kit (Solarbio, Beijing, China), and all the samples were filled to a uniform range and counted. The cell suspensions were inoculated into a 96-well plate with 1 × 10^6^ cells/well, and then the NA protein or ConA protein was added at a final concentration of 20 µg/mL and incubated for 72 h to stimulate lymphocyte proliferation. The stimulation indices were measured with a CCK-8 (Meilunbio, Dalian, China) to evaluate lymphocyte proliferation. The cell-mediated immune responses were further validated using flow cytometry. Specifically, the cell density was adjusted to 2.5 × 10^6^/well, and 5,6-carboxyfluorescein diacetate succinimidy ester (CFSE) (BD Bioscience) was added at a final concentration of 2.5 µg and incubated at 37°C for 10 min. The cells were then incubated with medium containing 20 µg/mL NA protein at 37°C for 72 h. Then, the cells were collected and incubated with mouse anti-chicken CD4 and CD8 antibodies (Southern Biotechnology, Birmingham, AL) to determine cell proliferation.

### Detection of cytokine expression

Spleen lymphocytes were prepared as described above and inoculated into a six-well plate at a density of 1 × 10^6^ cells/well. NA protein was added at a final concentration of 20 µg/mL and incubated for 72 h. Then, the cells were collected, and total RNA was extracted with a TransZol Up Plus RNA Kit (TransGen, Beijing, China) to determine IFN-γ and IL-4 mRNA levels using qRT-PCR. The primers used are shown in Table 3. The concentrations of IFN-γ and IL-4 in the supernatants were also determined using commercial ELISA kits (Kete, Jiangsu, China).

### Determination of lung virus titers and inflammatory cytokines after challenge

The lung tissue and trachea of the chickens were collected on day 5 after challenge, and 0.2 g of tissue sample was used to extract either total viral RNA using an RNeasy Viral RNA Isolation Kit (Beyotime, Shanghai, China) or total tissue RNA extraction by a TransZol Up Plus RNA Kit (TransGen, Beijing, China), respectively. Viral load and inflammatory cytokine (IL-6 and IL-1β) production were subsequently evaluated with qRT-PCR using the primers listed in Table 3.

### Viral shedding in chickens after challenge

Oropharyngeal swabs and cloacal swabs from the chickens were collected on the 3rd, 5th, 7th, and 9th days after challenge and placed in antibiotic solution (PBS, 100 U/mL penicillin-streptomycin, 50 µg/mL kanamycin, 50 µg/mL gentamicin, 50 µg/mL) to detect virus shedding in the chickens by inoculation into 9-day-old SPF chicken embryos, followed by a hemagglutination test.

### Statistical analysis

All the data are presented as the SEM ± mean. One-way analysis of variance or *t*-tests were used for statistical analysis in Prism 10.2.3 (GraphPad). *P* < 0.05 was considered to indicate statistical significance.

## RESULTS

### Construction of the tetramer-NA and tetramer-NA-DCpep plasmids

The extracellular region of the NA gene (32–466 aa) from the H9N2 AIV G57 serotype was ligated with the hVASP tetramer domain and inserted into the parental plasmid pYL46, with or without the chicken DCpep sequence, yielding pYL314 and pYL323, respectively (Fig. 1A). The successful expression of the NA protein was determined using both immunofluorescence (Fig. 1B) and western blot (Fig. 1C through F) assays using an NA protein-specific antibody. Transfected 293T cells were collected, and the intracellular production of the NA protein (Fig. 1D) was confirmed; GAPDH was also included as an internal control (Fig. 1C). The supernatants were also collected and subjected to denaturing western blotting, and the results revealed that NA proteins could be efficiently secreted by both pYL314 and pYL323 (Fig. 1E). Unexpectedly, the protein band in the supernatant of pYL314-transfected cells appeared to be slightly larger than that in the supernatant of pYL323-transfected cells. Similarly, the protein band in a native western blot analysis revealed that the protein size of the pYL314 supernatant was somewhat greater than that of pYL323 (Fig. 1F).

### Dendritic cell-targeted tetramer-NA protein moderately stimulates DC maturation *in vitro*

**Fig 2:**
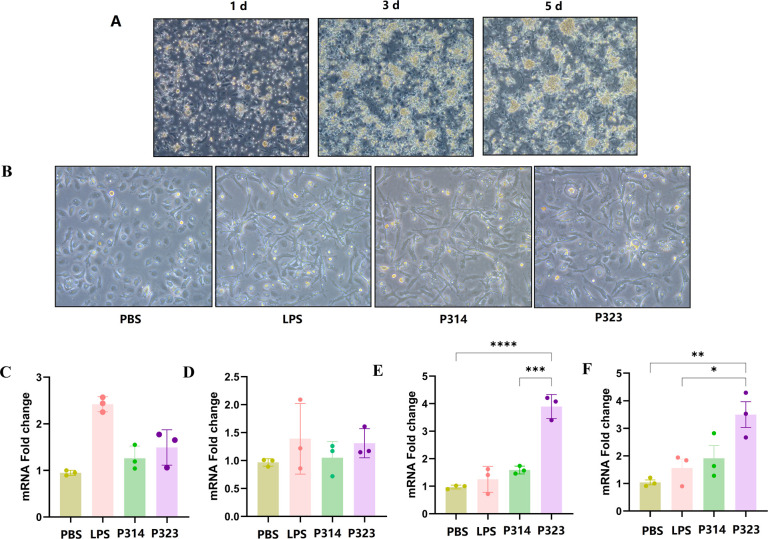
Maturation of chicken BMDCs stimulated with tetramer-NA proteins from pYL314 (P314) or pYL323 (P323). The chicken BMDCs were prepared as described in the Materials and Methods section, and the cell morphology on the 1st, 3rd, and 5th days was recorded (**A**). On the 7th day, the cells were incubated with P314 or P323 at a concentration of 20 µg/mL for 24 h, and the morphology was recorded (**B**). LPS (1 mg/mL) and PBS were used as positive and negative controls, respectively. Total RNA was subsequently extracted and analyzed using qRT-PCR for the expression of CCL5 (**C**), CCR7 (**D**), CD83 (**E**), and CD86 (**F**) (*n* = 3; *P < 0.05, **P < 0.01, ***P < 0.001, and ****P < 0.0001).

chBMDCs were prepared as described above, and their morphology was monitored daily, after which the morphology on the 1st, 3rd, and 5th days was recorded (Fig. 2A). After the 7th day, the cells were stimulated with P314 or P323 for 24 h, and LPS and PBS were used as positive and negative controls, respectively (Fig. 2B). Most of the cells in the negative control group that were stimulated with PBS were round or oval, with smooth surfaces and no protrusions, and there was no obvious change in the number of cells. The dendritic cells stimulated by LPS, P314, and P323 were clearly differentiated. The cells were irregular, with many dendritic protrusions on the surface, which were stellate or multipolar. In addition, the adherence was loose, and the cell volume increased significantly. However, compared with that in the positive control group, the degree of differentiation did not significantly differ between the P314 and P323 groups under light microscopy, and subsequent post-processing analysis of cell recovery was performed. The cells were subsequently collected and subjected to qRT-PCR to measure the expression of the markers CCL5 (Fig. 2C), CCR7 (Fig. 2D), CD83 (Fig. 2E), and CD86 (Fig. 2F). The results revealed that compared with P314, P323 increased CCL5 and CCR7 expression levels, but the difference was not statistically significant. However, compared with incubation with either LPS or P314, incubation with P323 significantly increased CD83 and CD86 mRNA levels, indicating that the presence of chDCpep could dramatically increase chBMDC maturation through the targeting of tetramer-NA to DCs.

### Biological characteristics of Δ*focA* attenuated *Salmonella*

**Fig 3:**
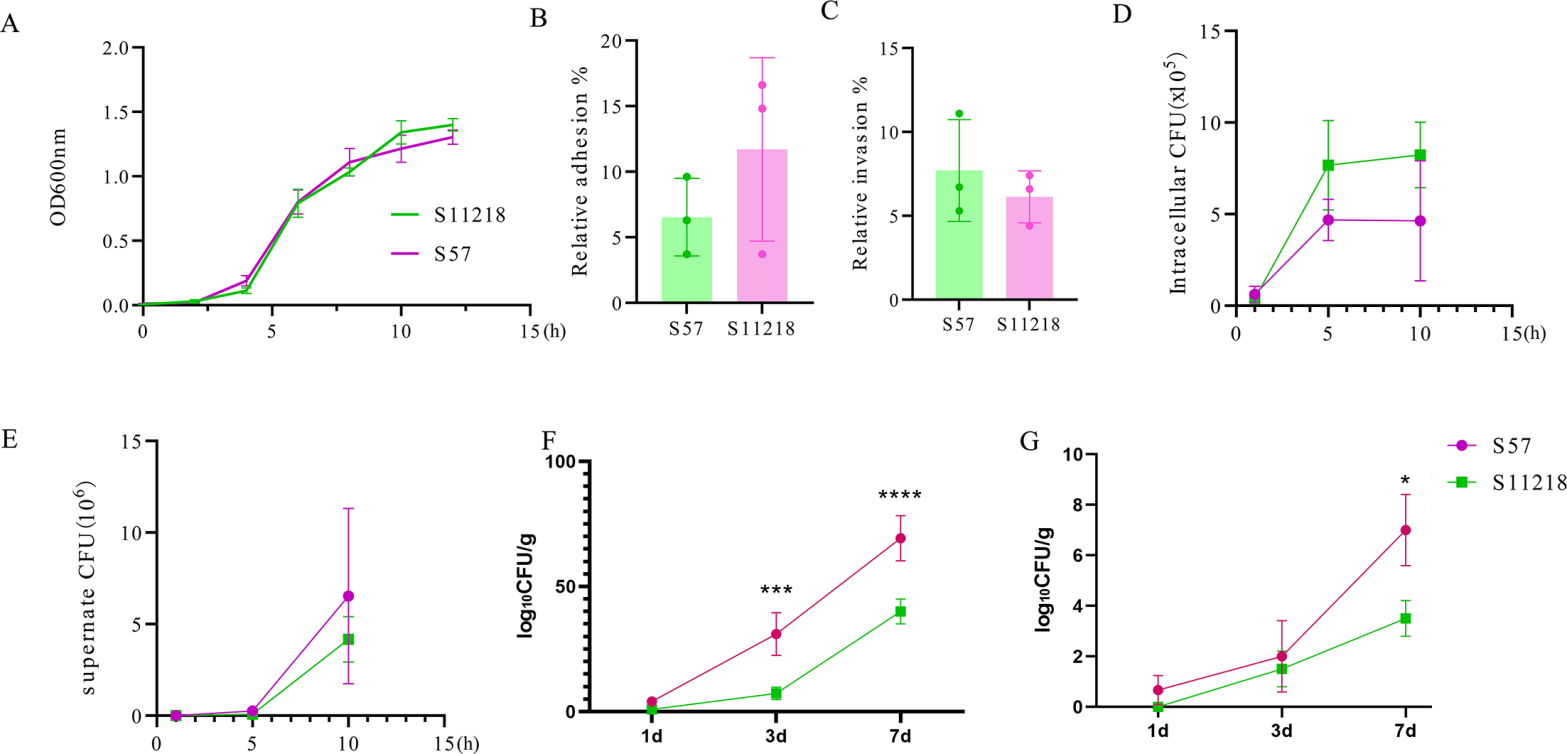
Effects on the physiological characteristics of the *focA*-deleted strain. The growth curves of the initial strain S11218 and the *focA* gene deletion strain S57 were measured (**A**). Both strains were inoculated into fresh LB medium supplemented with 0.2% arabinose and DAP and incubated at 37°C with shaking. The OD_600_ values of both cultures were recorded every 2 h until 12 h. Macrophage RAW264.7 cells were then incubated with both strains, and their adhesion (**B**), invasion (**C**), intracellular proliferation (**D**), and escape (**E**) abilities were investigated as described above. In addition, the colonization of *Salmonella* in the liver (**F**) and spleen (**G**) of chickens was determined on the 1st, 3rd, and 7th days post-immunization (*n* = 3; *P < 0.05, ***P < 0.001, and ****P < 0.0001, respectively).

The Δ*focA*-attenuated *Salmonella* strain was generated using a pRE112-based suicide vector and two rounds of selection as described above, and *focA* gene deletion was confirmed with PCR using the primers focA-UP-F2-SmaI/focA-DOWN-R2-XbaI (data not shown). The growth curve revealed no obvious difference between the mutant *Salmonella* χYL57 (S57) and the parental *Salmonella* χ11218 (S11218) during the 12 h culture (Fig. 3A). The ability of χYL57 to adhere to and invade macrophages was subsequently analyzed using *in vitro* experiments. The results revealed that the adhesion (Fig. 3B) and invasion (Fig. 3C) between these strains were similar, even though some differences could be observed; however, the difference was not statistically significant. The intracellular survival (Fig. 3D) and number of escaped bacteria in the supernatants (Fig. 3E) were also determined after 10 h of incubation. Although the *focA* gene deletion did not dramatically affect either intracellular survival (Fig. 3D) or bacterial numbers in the supernatants (Fig. 3E), the *focA* gene deletion mutation tended to increase cell escape ability; however, further studies are needed to confirm this phenomenon. In addition, an *in vivo* study was performed to evaluate the effect of *focA* gene deletion on *Salmonella* colonization in chicken liver (Fig. 3F) and spleen (Fig. 3G) on days 1, 3, and 7 after oral immunization. Compared with the parental S11218 strain, *Salmonella* with the *focA* gene deletion could colonize more efficiently in both the liver and the spleen, especially in the chicken liver. To further address the safety concern associated with *focA* gene deletion, liver samples after oral immunization were collected from 3-day-old and 7-day-old chickens and subjected to hematoxylin-eosin (HE) staining, and the results indicated no obvious inflammatory infiltration after immunization with either strain (Fig. S2).

### The combination of DC-targeting design and *focA* mutation efficiently increased humoral, mucosal, and cellular immune responses in chickens

**Fig 4:**
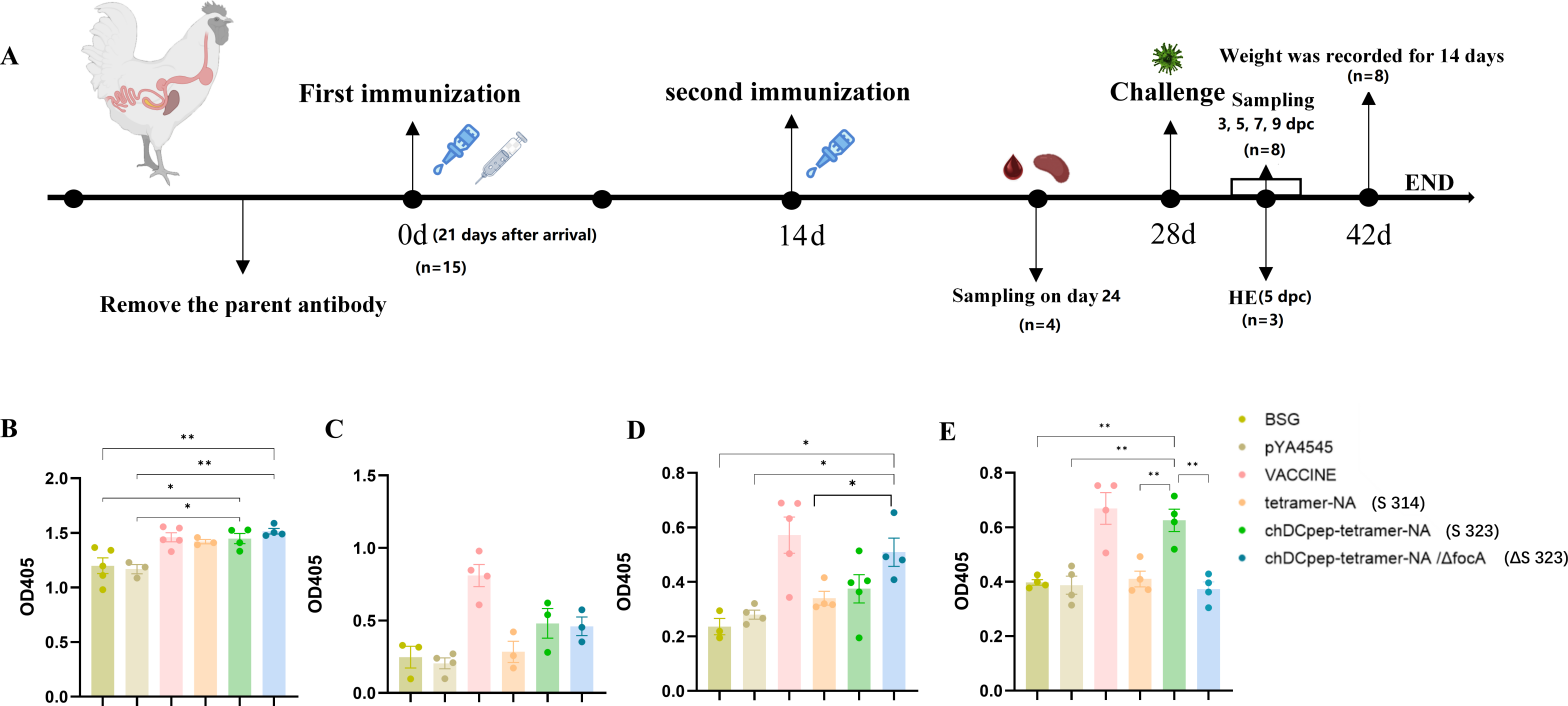
Chicken experimental design and NA-specific antibody production. Chickens were immunized first on day 21 after arrival to avoid the effects of parental antibodies, which was recorded as day 0. A second boost immunization was performed 14 days later. Samples were collected on day 24 (10 days after the second immunization, 10 dp2i) for antibody detection, cell proliferation, and the intracellular production of cytokines. A viral challenge was performed on day 28, and the cells were monitored for 14 days. Oropharyngeal and cloacal swabs were collected and inoculated into 10-day-old chicken embryos to detect virus shedding on days 3, 5, 7, and 9 post-viral challenge (dpc) (**A**). IgG in serum (**B**), IgA in lung washing (**C**), tracheal washing (**D**), and intestinal washing samples (**E**) were collected and detected using ELISA to evaluate the binding antibody titers (*P < 0.05, **P < 0.01).

A chicken experiment was designed as shown (Fig. 4A), and samples were collected at 10 dp2i for analysis of NA-specific binding antibodies in serum (Fig. 4B), bronchoalveolar lavage (Fig. 4C), tracheal lavage (Fig. 4D), and intestinal washing (Fig. 4E) using ELISA. In general, compared with the other groups, the tetramer-NA-DCpep plasmid with the parental strain χ11218 (S323) or the *focA* mutant χYL57 strain (ΔS323) elicited the highest antibody titers in all the samples, except for the commercial vaccine group, indicating that the presence of chicken DC-targeting peptides could benefit humoral and mucosal immune responses regardless of whether the *focA* mutation was present. In detail, the *Salmonella* strain with the *focA* deletion exhibited increased sIgA production in tracheal lavage (Fig. 4D), whereas the parental *Salmonella* appeared to be better at inducing sIgA in intestinal washing samples (Fig. 4E). Notably, although the inactivated vaccine was not efficient at inducing a mucosal immune response, our results demonstrated that the commercial inactivated vaccine could also stimulate the generation of an sIgA antibody response (Fig. 4C through E).

**Fig 5:**
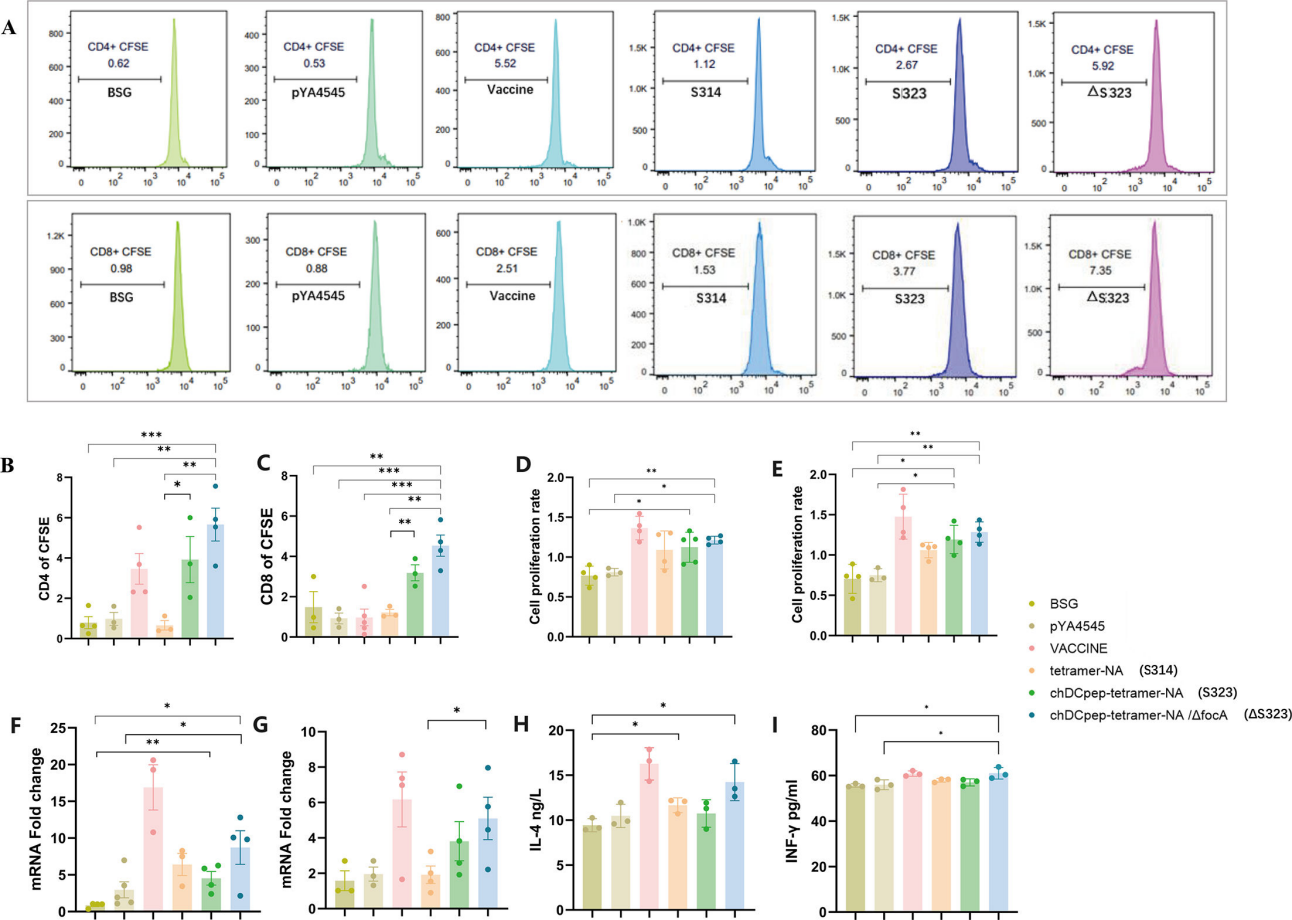
NA protein stimulates chicken spleen lymphocyte proliferation and the production of intracellular cytokines. Flow cytometry (**A**) was performed to determine the proliferation of CD4^+^ T cells (**B**) and CD8^+^ T cells (**C**) among spleen cells stimulated with the NA protein. A CCK-8 assay was also conducted using the NA protein (**D**) and ConA (**E**) to determine cell proliferation. In addition, the spleen lymphocytes were stimulated with NA proteins, and intracellular IL-4 (**F**) and IFN-γ (**G**) mRNA levels as well as IL-4 (**H**) and IFN-γ (**I**) concentrations were measured using qRT-PCR or ELISA, respectively (*n* = 5; *P < 0.05, **P < 0.01, and ***P < 0.001).

Cell proliferation was determined using both a CFSE labeling assay with flow cytometry (Fig. 5A through C) and a CCK-8 assay (Fig. 5D and E). The results showed that the tetramer-NA-chDCpep plasmid delivered by both *Salmonella* strains could effectively stimulate the proliferation of CD4 and CD8 spleen lymphocytes, especially for the *focA*-deleted *Salmonella* and CD8 T cells (Fig. 5C). Similar trends were also observed using CCK-8 analysis, although somewhat different results were observed (Fig. 5D and E), indicating that the presence of a chicken DC-targeting peptide could dramatically stimulate T cell proliferation, whereas the *focA* mutation could further increase this effect.

IL-4 and IFN-γ mRNA levels in spleen cells stimulated with the NA protein were then determined using qRT-PCR. In general, the combination of a chicken DC-targeting peptide and the *focA* mutation dramatically increased the mRNA levels of both IL-4 (Fig. 5F) and IFN-γ (Fig. 5G), whereas the single use of a DC-targeting design did not. Similar trends were also observed when ELISA was used to evaluate IL-4 (Fig. 5H) and IFN-γ (Fig. 5I) levels in cell supernatants.

### Protection against virus challenge assessed based on lung lesions and histopathological observations

**Fig 6:**
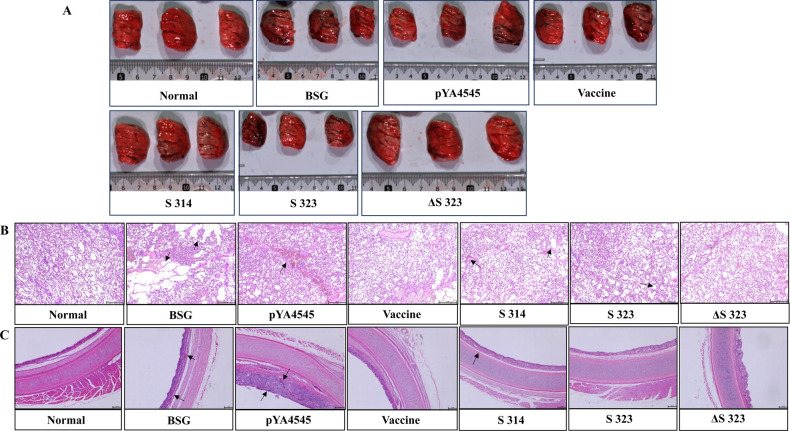
Morphological and histopathological analysis of lung and tracheal samples. At 5 days post-challenge, the chickens were necropsied, and lung and tracheal samples were collected. Then, the morphological changes in the lungs were determined (**A**). After that, both lung (**B**) and tracheal (**C**) samples were subjected to HE staining. Evaluation was performed by the naked eye, and body weight was monitored after the challenge.

As shown in Fig. 6A, compared with that of the nonchallenged group (normal), the lung tissue of the BSG group and the empty vector group (pYA4545) showed obvious morphological atrophy, and white foam-like liquid exudation was observed when the cells were gently pressed with fingers. The lung tissue of the empty vector group and the S314 group showed obvious necrosis at the edge. The lung tissue of one chicken in the S323 group showed necrosis, whereas the morphological changes in the ΔS323 group were similar to those in the normal group. Histopathological lung tissue and tracheal tissue sections were also analyzed. The lung tissue results (Fig. 6B) revealed that compared with the normal group, the BSG group had a large area of compensatory emphysema, and the BSG group and the empty vector group showed extensive inflammatory cell infiltration and visible purulent exudate in the trachea. Compared with the vaccine group, the lung tissue of the empty vector group showed obvious alveolar wall thickening. The same phenomenon occurred in the S323 group, albeit to a lesser extent. Both the S314 group and the ΔS323 group showed mild bronchial congestion, which was accompanied by local emphysema and a small amount of inflammatory cell infiltration; however, these phenomena were not observed in the ΔS323 group. Histopathological results of tracheal tissue (Fig. 6C) revealed that compared with the normal group, the BSG group and the empty vector group exhibited severe inflammatory cell infiltration and blood cell infiltration in mucosal epithelial cells. In addition, epithelial cell enlargement and mucosal shedding occurred. The S314 group and the S323 group were similar, but the effects were less notable. Compared with the vaccine group and the normal group, the cilia on the surface of the epithelial cells in the ΔS323 group were not arranged neatly, and the mucosal cells were loosely arranged. However, there was no obvious inflammation.

### Viral load and inflammatory factor levels in the lungs and trachea

**Fig 7:**
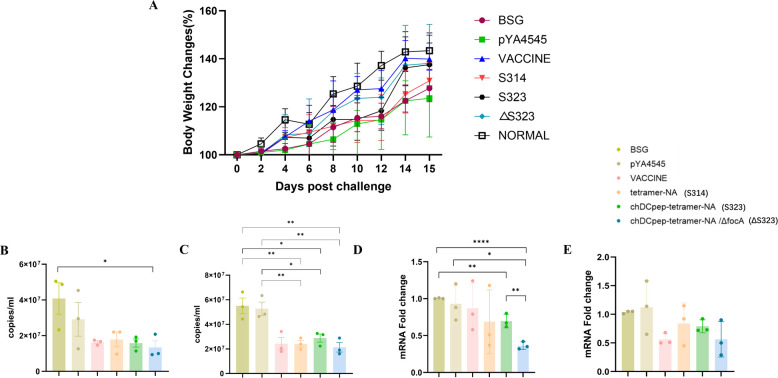
Body weight gain and virus loading after challenge**.** Body weight gain was recorded for 14 days after the challenge (**A**). The virus titers in lung (**B**) and tracheal (**C**) samples were subsequently measured using qRT-PCR. In addition, the mRNA levels of the inflammatory cytokines IL-6 (**D**) and IL-1β (**E**) in lung samples were also evaluated using qRT-PCR (*n* = 3; *P < 0.05, **P < 0.01, and ****P < 0.0001).

The body weight gains of the chickens after challenge were also monitored for 14 days, and the results indicated that the weights of all the experimental groups significantly fluctuated after challenge and began to notably increase on the 5th day, after which they tended to stabilize on the 13th day (Fig. 7A). Notably, the body weight gain in the ΔS323 group appeared to be very similar to that in the inactivated vaccine group after the 7th day post-challenge and was clearly better than that in the other groups. However, the presence of chDCpep alone did not yield the same results, indicating that the combination of the *focA* mutation and chDCpep was necessary for our vaccine design.

The viral load and levels of inflammatory factors in the lungs and trachea of the chickens in each group following viral infection were also measured. The results revealed that the viral load in both lung (Fig. 7B) and trachea (Fig. 7C) samples from all the experimental groups dramatically decreased compared with that in the BSG control and empty vector groups. Specifically, the viral load in the ΔS323 group was greater than that in the other groups. Similar results were also observed for the quantification of the mRNA levels of the inflammatory cytokines IL-6 (Fig. 7D) and IL-1β (Fig. 7E) in lung tissues. Although all the *Salmonella*-vectored strains decreased the production of inflammatory cytokines, oral immunization with ΔS323 performed the best, especially with respect to IL-6 levels, performing even better than the inactivated vaccine. These results indicate that the combination of a DC-targeting strategy and *focA* mutation is necessary for enhanced protection.

The degree of viral shedding was then analyzed by detecting the collected oropharyngeal swabs and cloacal swabs (Table 4). Viral shedding was detected in both oropharyngeal swabs and cloacal swabs at all time points in the BSG and empty vector pYA4545 groups. Notably, the commercial inactivated vaccine was still the most effective group for preventing virus shedding. In addition, viral shedding could only be observed on days 3 and 5 in oropharyngeal swabs but not on the other days or in cloacal swabs. On the other hand, compared with the negative control, all the *Salmonella*-infected groups exhibited alleviated viral shedding in both swabs, yielding dramatically decreased viral shedding. Notably, compared with S314 and S323, immunization with ΔS323 decreased the rate of positive viral shedding in oropharyngeal swabs at days 3 and 5 post-challenge. In addition, the DC-targeting S323 group appeared to be better than the nontargeting S314 group, even though it was not as good as the ΔS323 group, indicating that the use of a DC-targeting approach still benefits vaccine design.

**TABLE 4:** Table detox after challenge AIV

Groups	Oropharyngeal swab				Cloacal swab			
	Days after challenge AIV							
	3	5	7	9	3	5	7	9
BSG	7/8	7/8	2/8	1/8	2/8	1/8	1/8	1/8
pYL4545	7/8	7/8	2/8	1/8	2/8	1/8	1/8	1/8
Vaccine	6/8	3/8	0/8	0/8	0/8	0/8	0/8	0/8
S314	8/8	7/8	2/8	0/8	1/8	0/8	0/8	0/8
S323	5/8	6/8	2/8	0/8	1/8	1/8	0/8	0/8
ΔS323	4/8	5/8	1/8	0/8	1/8	1/8	0/8	0/8

## DISCUSSION

Currently, the low-pathogenicity H9N2 influenza virus is the predominant subtype circulating in Asian countries. Although vaccination remains one of the primary strategies to control H9N2 subtype AI in China, the majority of vaccine recipients are still under the siege of wild-type variants. Therefore, disease outbreaks still occur in vaccinated flocks in the case of decreased protection levels or different types of immune failure (31). There is an urgent need for the development of next-generation influenza vaccines capable of inducing broad-spectrum protective efficacy. Notably, the NA protein of influenza has recently been identified as an ideal vaccine candidate; in particular, the formation of a tetrameric NA head is considered critical for an efficient AIV vaccine (16). Most recent studies on NA immune responses have been performed with secreted rNA chimeras that use a stabilizing tetramerization domain as a functional surrogate for the transmembrane domain (32). To improve vaccine efficiency, the tetramer-NA could be conjugated on the surface of nanoparticles, such as virus-like particles, to increase the immunogenicity of soluble recombinant antigens (32).

In this study, a tetramerization domain, hVASP (17), was designed to ligate the extracellular region of the NA protein to yield a tetrameric NA antigen; in addition, chicken DCpep was included to improve the humoral immune response. However, NA and NA-DCpep protein synthesis appeared to differ from our original expectations according to the western blot results. Although the protein size in cells transfected with pYL314 and pYL323 was similar (Fig. 1D), the protein size in the supernatants of pYL314-transfected cells was dramatically greater than that in the supernatants of pYL323-transfected cells, as determined by both denaturing (Fig. 1E) and native (Fig. 1F) western blotting. One possible explanation is that during the translocation of the NA protein outside the transfected cells, the secretion signal peptide was affected by the conformation of the polymer NA proteins, which resulted in inefficient separation of the signal peptide and NA protein. However, the presence of chDCpep could alleviate these side effects, yielding a smaller NA-DCpep protein band. Further studies, such as cryo-transmission electron microscope (TEM), will be necessary to determine the underlying mechanisms involved. Interestingly, the protein size of the “designed tetramer” in both the pYL314 and pYL323 groups was not as expected to form a “real tetramer” (Fig. 1F). The protein size in both groups appeared more likely to yield a “dimer tetramer” or even a “double dimer tetramer.” One of the possible reasons for these results is that the extracellular region of NA in this study was the 32–466 aa region, which included the Cys residue at position 49 in the stalk region of the NA protein that could form an intermolecular disulfide bond (33). Therefore, further study with a shorter stalk region of the NA excluding the 49-Cys residue should be beneficial for the correct formation of the expected “tetramer conformation.” Notably, although the conformation of either tetramer-NA or tetramer-NA-DCpep was not as expected, because the presence of DCpep still efficiently stimulated the maturation of chicken BMDCs *in vitro* (Fig. 2E and F), we will continue to perform our animal studies later.

The *focA* gene is an important virulence factor in *Salmonella* enteritidis, and the loss of the *focA* gene could increase the ability of *Salmonella* to exit macrophages and increase early extraintestinal spread for systemic infection in a mouse model (34). Inspired by these results, we constructed a *focA* gene mutation in our original regulated delayed-lysis *Salmonella* strain to determine whether the mutation could help to improve vaccine efficiency because it would benefit the spread of the *Salmonella* strain. Similar decreases in the number of intracellular bacteria and increases in macrophage escape were also observed *in vitro* (Fig. 3D and E). More importantly, the *focA*-deficient *Salmonella* strain appeared to more efficiently colonize both the spleen and liver (Fig. 3F and G), indicating its potential ability to enhance the humoral immune response. On the other hand, the spleen and liver samples were also subjected to histological staining to exclude any possible side effects on chicken health. The results revealed no obvious difference between the two groups (data not shown), which is partially inconsistent with the findings of a previous report indicating that pyroptosis and apoptosis ability were not altered due to the *focA* deletion in mice (34). However, further studies are needed to address safety concerns, such as the production of proinflammatory cytokines in the spleen and liver.

The inclusion of DC-targeting peptides or antibodies has been proven to be an interesting and efficient approach to elicit enhanced humoral immune responses, especially cellular immune responses, in both mammalian and chicken studies (35–37). Our results were at least partially consistent with previous observations regarding the use of a chicken DC-targeting peptide. The presence of chDCpep significantly increased the generation of NA-specific IgA antibodies in intestinal samples (Fig. 4E) and CD4 and CD8 T-cell proliferation in the spleen (Fig. 5B and C). However, the presence of chDCpep alone did not dramatically affect the other results measured in this study. On the other hand, compared with chDCpep alone, the combination of the *focA* mutation and chDCpep appeared to increase T cell proliferation (Fig. 5B and C) and intracellular IL-4 and IFN-γ mRNA levels (Fig. 5F and G). In addition, the presence of the *focA* mutation significantly decreased the production of the inflammatory cytokine IL-6 (Fig. 7D), providing better protection against viral challenge by maintaining body weight gain (Fig. 7A) and less severe histopathological changes (Fig. 6). With respect to viral shedding, although oral administration of ΔS323 did not completely prevent viral shedding from oropharyngeal and cloacal swabs, the combination of chDCpep and the *focA* mutation could increase protection by decreasing the percentage of virus-positive cells compared with that in other groups, indicating its potential application in vaccine design.

## Data Availability

The data that support the findings of this study are available from the corresponding author upon reasonable request.

## SUPPLEMENTAL MATERIAL

Supplemental figures spectrum.01421-25-s0001.docx Figures S1 and S2.

ASM does not own the copyrights to Supplemental Material that may be linked to, or accessed through, an article. The authors have granted ASM a non-exclusive, world-wide license to publish the Supplemental Material files. Please contact the corresponding author directly for reuse.
