# Supplementary material for: Protection against H9N2 challenge in chickens via Salmonella with a focA mutation delivered by a dendritic cell-targeted avian influenza virus NA protein tetramer
Source: Microbiol Spectr. 2025 Oct 27;13(12):e01421-25. doi: 10.1128/spectrum.01421-25 (PMC12671097; doi:10.1128/spectrum.01421-25)
Supplement: Supplemental figures — Figures S1 and S2. [file spectrum.01421-25-s0001.docx]

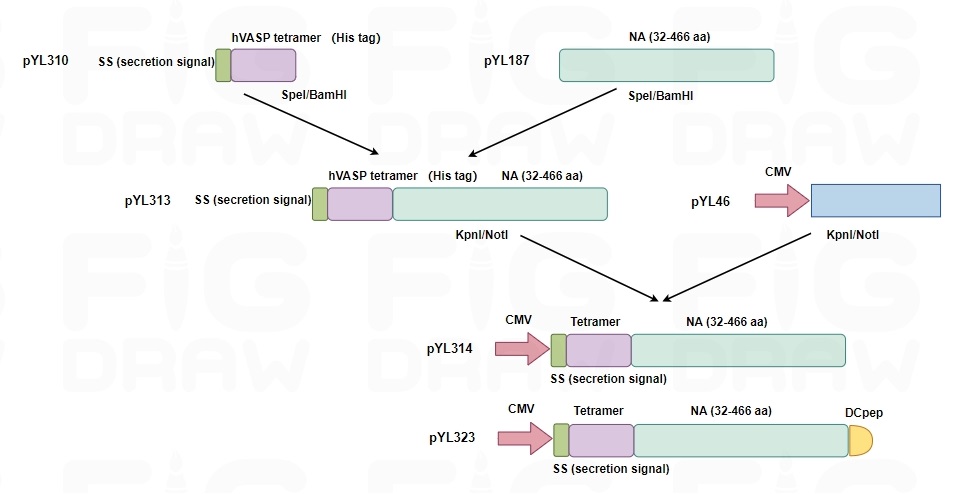


**Sup Fig. 1 Illustration of plasmid construction.**


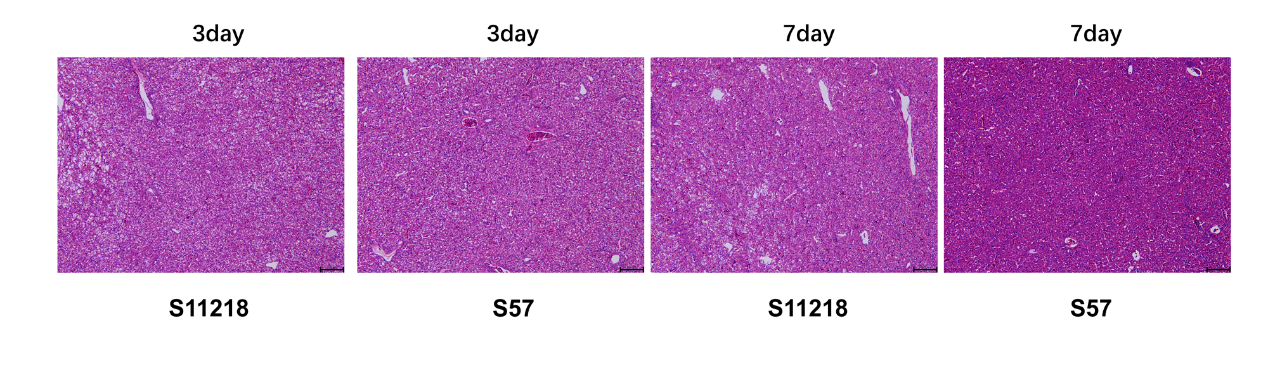


**Sup Fig. 2 HE staining of chicken liver samples after immunization with both wild-type *Salmonella* and the *focA* gene deletion mutation.**
